# Methionine adenosyltransferase α2 sumoylation positively regulate Bcl-2 expression in human colon and liver cancer cells

**DOI:** 10.18632/oncotarget.5342

**Published:** 2015-09-25

**Authors:** Maria Lauda Tomasi, Minjung Ryoo, Komal Ramani, Ivan Tomasi, Pasquale Giordano, José M. Mato, Shelly C. Lu

**Affiliations:** ^1^ Division of Gastroenterology, Cedars-Sinai Medical Center, Los Angeles, CA 90048, USA; ^2^ USC Research Center for Liver Diseases, Keck School of Medicine of University of Southern California, Los Angeles, CA 90033, USA; ^3^ General Surgery Department, Rotherham Hospital, Rotherham S60 2UD, UK; ^4^ Colorectal Department, Barts Health, London E11 1NR, UK; ^5^ CIC bioGUNE, Centro de Investigación Biomédica en Red de Enfermedades Hepáticas y Digestivas (Ciberehd), Technology, Park of Bizkaia, 48160 Derio, Bizkaia, Spain

**Keywords:** methionine adenosyltransferase α2, Bcl-2, ubiquitin-conjugating enzyme 9, sumoylation, colon cancer

## Abstract

Ubiquitin-conjugating enzyme 9 (Ubc9) is required for sumoylation and inhibits apoptosis via Bcl-2 by unknown mechanism. Methionine adenosyltransferase 2A (*MAT2A*) encodes for MATα2, the catalytic subunit of the MATII isoenzyme that synthesizes S-adenosylmethionine (SAMe). Ubc9, Bcl-2 and MAT2A expression are up-regulated in several malignancies. Exogenous SAMe decreases Ubc9 and MAT2A expression and is pro-apoptotic in liver and colon cancer cells. Here we investigated whether there is interplay between Ubc9, MAT2A and Bcl-2. We used human colon and liver cancer cell lines RKO and HepG2, respectively, and confirmed key finding in colon cancer specimens. We found MATα2 can regulate Bcl-2 expression at multiple levels. MATα2 binds to *Bcl-2* promoter to activate its transcription. This effect is independent of SAMe as MATα2 catalytic mutant was also effective. MATα2 also directly interacts with Bcl-2 to enhance its protein stability. MATα2's effect on Bcl-2 requires Ubc9 as MATα2's stability is influenced by sumoylation at K340, K372 and K394. Overexpressing wild type (but not less stable MATα2 sumoylation mutants) protected from 5-fluorouracil-induced apoptosis in both colon and liver cancer cells. Colon cancer have higher levels of sumoylated MATα2, total MATα2, Ubc9 and Bcl-2 and higher MATα2 binding to the *Bcl-2* P2 promoter. Taken together, Ubc9's protective effect on apoptosis may be mediated at least in part by sumoylating and stabilizing MATα2 protein, which in turn positively maintains Bcl-2 expression. These interactions feed forward to further enhance growth and survival of the cancer cell.

## INTRODUCTION

SUMO is a small ubiquitin-like protein that can be covalently attached to proteins through the formation of isopeptide bonds with specific lysine residues of target proteins [1]. Four SUMO family members (SUMO-1 to -4) are encoded by distinct genes in mammals [2]. SUMO-1 regulates protein stability and activity with crucial implications for many cellular pathways [3]. In contrast, protein conjugates with heterologous SUMO-2/3-ubiquitin chains are preferentially targeted for proteasome degradation and the function of SUMO-4 is unknown [4–6]. Sumoylation is a multiple-step process, involving maturation, activation, conjugation and ligation [1]. Ubiquitin conjugating enzyme 9 (Ubc9) is the only E2 conjugating enzyme and therefore a key regulator of the sumoylation machinery, transferring the activated SUMO to protein substrates [7]. Sumoylation is involved in many vital processes including transcriptional regulation, signal transduction, protein degradation, cell cycle regulation, chromatin organization, and nuclear transport [8]. Dysregulated sumoylation contributes to carcinogenesis by affecting post-transcriptional modification of key proteins [9], including those involved in cancer metastasis [10–12].

Ubc9 has been shown to be a positive regulator of B-cell lymphoma 2 (Bcl-2) expression in breast cancer cell line MCF-7 [13]. Furthermore, higher rate of apoptosis and poor survival in the MCF-7 cells expressing dominant negative Ubc9 were associated with down-regulation of Bcl-2 [14]. Effect of Ubc9 on Bcl-2 expression was thought to be mediated by the estrogen receptor in MCF-7 cells [13], but the exact mechanism was not explored. Bcl-2 was identified first as an apoptotic regulator, the oncoprotein activated via chromosome translocation in human follicular lymphoma [15]. Bcl-2 acts by promoting cell survival rather than by driving cell proliferation as critical step in tumor development [15].

Methionine adenosyltransferase (MAT) is an essential cellular enzyme that catalyzes the formation of S-adenosylmethionine (SAMe), the principal biological methyl donor [16]. In mammals, this essential enzyme is the product of two different genes, *MAT1A* and *MAT2A*, which display a distinct pattern of expression among different tissues. *MAT1A* encodes for α1 that forms dimer (MATIII) and tetramer (MATI) that are predominantly expressed in liver parenchymal cells; while *MAT2A* encodes the α2 catalytic subunit of the MATII isoenzyme that is expressed in all other tissues [16]. Human liver and colon cancers have higher MAT2A expression [17–19], which is essential for growth as silencing MAT2A by sequence-specific small interfering RNA (siRNA) inhibited growth and induced apoptosis [19, 20]. We reported that SAMe treatment lowered Ubc9 protein expression and sumoylation in liver, colon and breast cancer cell lines [10]. We also reported SAMe treatment lowered MAT2A expression and is pro-apoptotic in liver and colon cancer cell lines [17, 20]. Since SAMe lowers the expression of both Ubc9 and MAT2A and knockdown of Ubc9 and MAT2A leads to apoptosis, we examined whether there might be interplay between MAT2A and Bcl-2 that is regulated by sumoylation. In the course of this work, we uncovered highly novel aspects of MATα2 function, namely the ability of MATα2 to regulate Bcl-2 expression by transcriptional and post-translational mechanisms that is modulated by sumoylation.

## RESULTS

### Effects of SAMe and methylthioadenosine (MTA) on Bcl-2 expression in HepG2 and RKO cells

**Figure 1:**
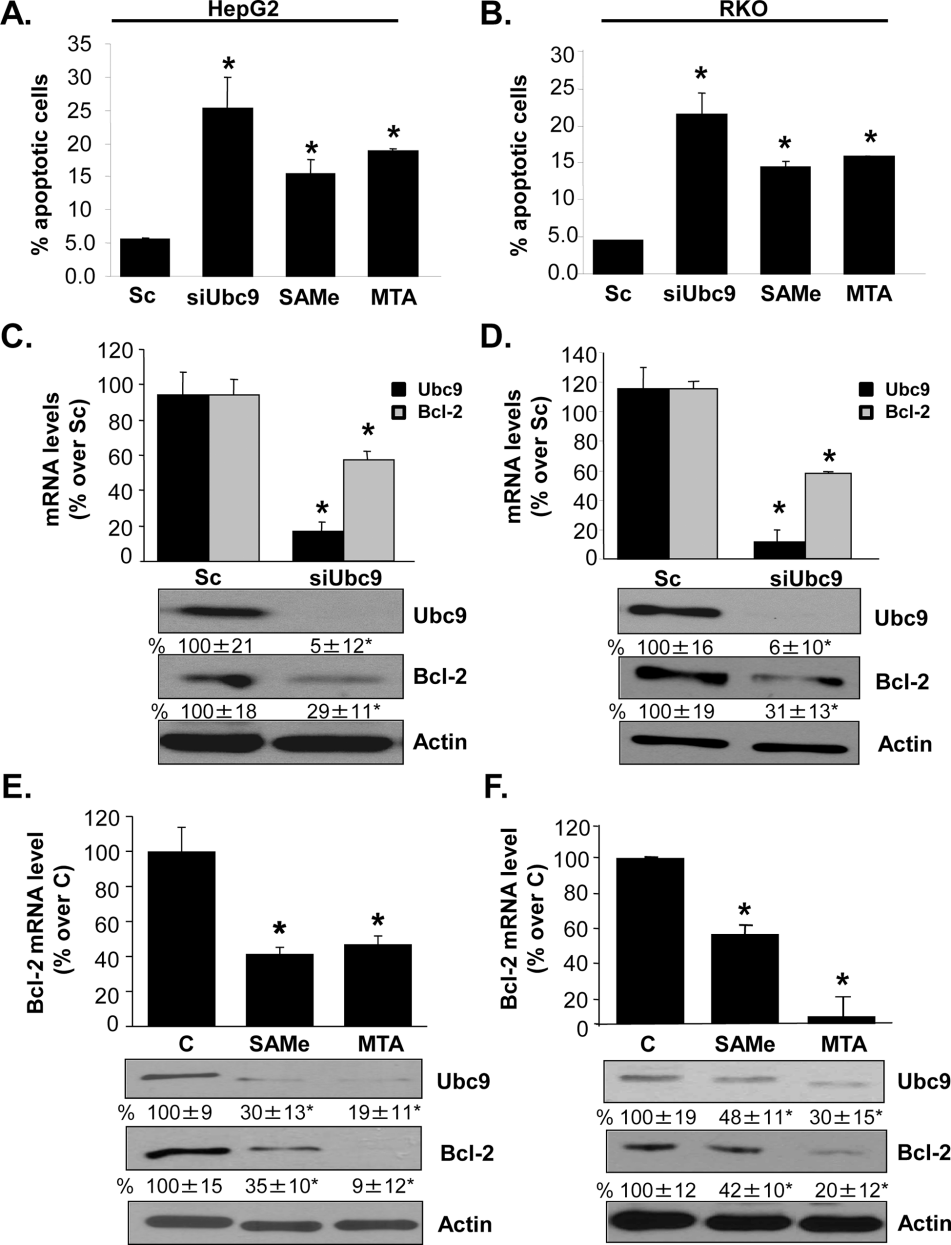
Ubc9 knockdown, SAMe and MTA treatment induce apoptosis and lower Bcl-2 expression in HepG2 and RKO cells HepG2 and RKO cells were treated with siUbc9 or scrambled siRNA (Sc, 20 nM) for 48 hours or SAMe (2 mM) and MTA (1 mM) for 24 hours. **A.** and **B.** Apoptosis was analyzed by nuclei Hoechst staining. Results from 3 experiments are shown as mean % of apoptotic cells ± SEM, *p < 0.001 vs Sc. **C–F.** Bcl-2 expression was determined by real-time PCR and Western blotting. Bcl-2 mRNA results are expressed as mean % of Sc or control ± SEM from 3 to 4 independent experiments performed in duplicate. *p < 0.001 vs. Sc for C and D; *p < 0.001 vs. control (C) for E and F. Densitometric changes in protein levels are shown below the blots. Results are expressed as mean % of Sc or control from 3 independent experiments ± SEM, *p < 0.02 vs. Sc for C and D, *p < 0.04 vs. control for E and F.

We previously reported that treatment with SAMe and its metabolite MTA induced apoptosis in HepG2 and RKO cells [19, 20] and lowered Ubc9 protein stability [10]. Ubc9 has been shown to regulate apoptosis as a positive regulator of Bcl-2 expression in breast cancer MCF-7 cells [14]. We next examined whether Ubc9 also regulate apoptosis and Bcl-2 expression in HepG2 and RKO cells. Treatment of HepG2 and RKO cells with Ubc9 siRNA (siUbc9) for 48 hours, or SAMe (2 mM) or MTA (1 mM) for 24 hours increased % apoptosis more than 5-, 3- and 3.5-fold, respectively (Figure 1A–1B). In both HepG2 and RKO cells, knockdown of *Ubc9* lowered Bcl-2 mRNA level after 48 hours by 39% and 40% (Figure 1C–1D), respectively. However, Western blot analysis shows the Bcl-2 protein level decreased by ~70% in both of cell lines (Figure 1C–1D). SAMe and MTA treatment also reduced Bcl-2 mRNA and protein levels (Figure 1E–1F).

### Effect of MAT2A silencing on Bcl-2 expression in HepG2 and RKO cells

**Figure 2:**
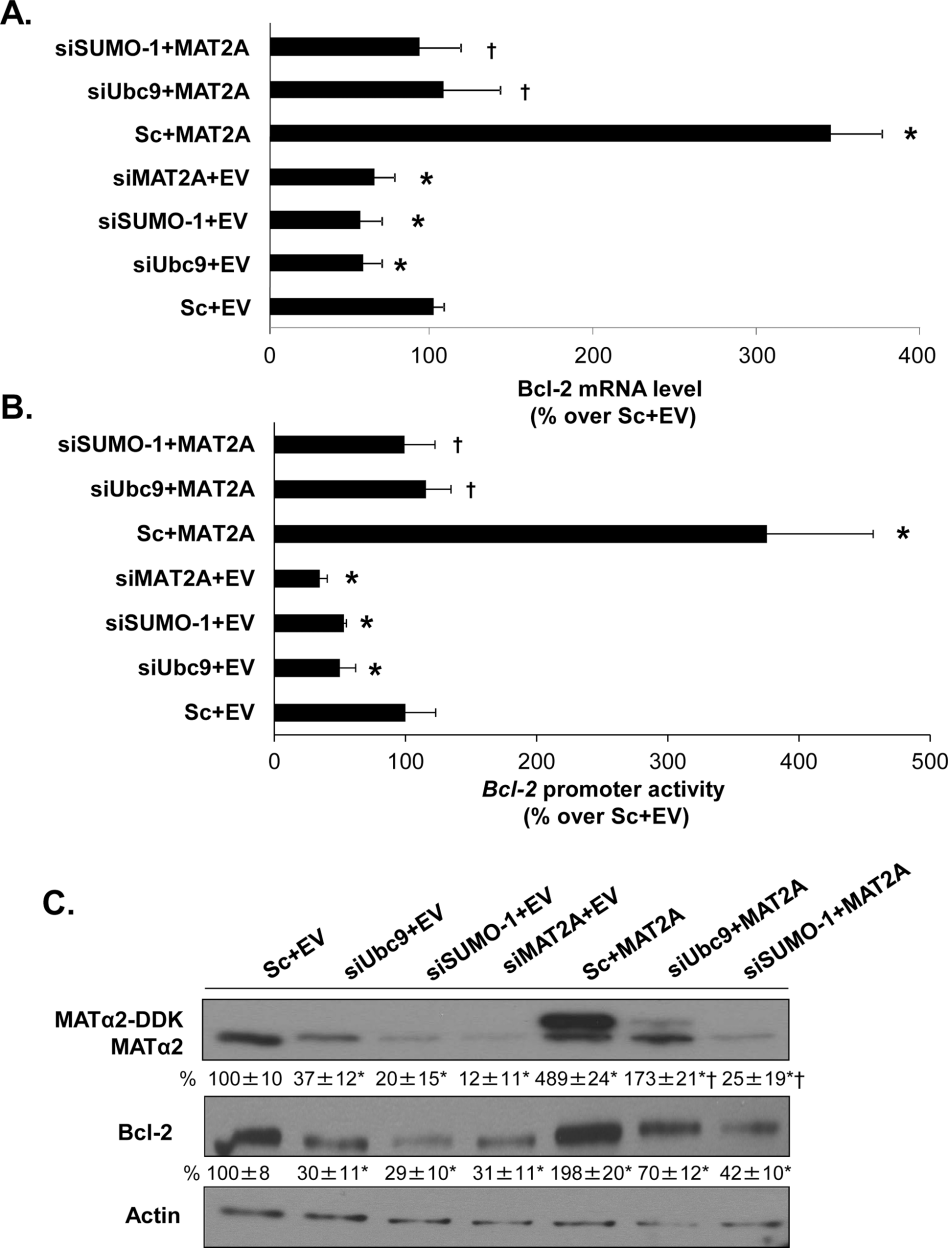
MAT2A and sumoylation machinery regulate Bcl-2 expression in HepG2 cells **A.** HepG2 cells were transfected with siMAT2A, siUbc9 and siSUMO-1 (20 nM) or scrambled siRNA (Sc) for 48 hours and/or MAT2A overexpression vector or empty vector (EV) for 24 hours. The mRNA levels of Bcl-2 were compared to Sc+EV using real-time PCR. Results represent mean ± SEM from 4 experiments in duplicates, *p < 0.04 vs. Sc+EV control; †*p* < 0.04 vs. Sc+MAT2A overexpression vector. **B.** Following the same treatments as in “A”, HepG2 cells were transfected with the human *Bcl-2* promoter or pGL3-basic as described in Methods. The luciferase activity driven by *Bcl-2* promoter was normalized to that of pGL3-Basic and expressed as % over Sc+EV. Results represent mean ± SEM from 4 experiments in duplicate, *p < 0.04 vs. Sc+EV control; †*p* < 0.03 vs. Sc+MAT2A overexpression vector. **C.** Cells were treated as in ‘A’ and total cellular protein was subjected to Western blotting with antibodies against MATα2 or Bcl-2. Densitometric changes were normalized to actin. Representative images and densitometric analysis (% mean ± SEM of Sc+EV, indicated below the blots) from 3 experiments in HepG2 cells are shown. *p < 0.05 vs. Sc+EV, †*p* < 0.03 vs. Sc+MAT2A overexpression vector.

Bcl-2 protein has well-known anti-apoptotic functions [21, 22]. In addition to lowering Ubc9 and Bcl-2 expression, SAMe and MTA treatment also lowered MAT2A expression [20] and knockdown of MAT2A in HepG2 and RKO cells induced apoptosis [17, 19]. These observations prompted us to examine whether there is interplay between MAT2A, Ubc9 and Bcl2. We used a gene silencing and overexpression of MAT2A approach in combination with siUbc9 or siSUMO-1 for 48 hours in HepG2 and RKO cells. Knockdown of MAT2A resulted in a 45% and 50% reduction in Bcl-2 mRNA level compared to a negative control siRNA, respectively, similar to the effects of siUbc9 and siSUMO-1 treatments (Figure 2A and Supplementary Figure S1A). Interestingly, overexpression of MAT2A increased Bcl-2 mRNA level by 3.3- and 3.4-fold as compared empty vector control and this inductive effect was largely eliminated if cells were also treated with siUbc9 or siSUMO-1 (Figure 2A and Supplementary Figure S1A). We next examined the *Bcl-2* promoter activity under the same experimental conditions in HepG2 and RKO cells. Figure 2B and Supplementary Figure S1B show that *Bcl-2* promoter activity highly correlated with the mRNA level results; specifically, knockdown of MAT2A, Ubc9 or SUMO-1 all lowered *Bcl-2* promoter activity, while overexpression of MAT2A increased *Bcl-2* promoter activity but not if either Ubc9 or SUMO-1 was knocked down (Figure 2B and Supplementary Figure S1B). Figures 2C and Supplementary Figure S1C show Western blot analyses under the same experimental conditions. MAT2A, Ubc9 or SUMO-1 knockdown lowered Bcl-2 protein level even more than mRNA level, whereas MAT2A overexpression raised Bcl-2 protein level but not if cells were also treated with either siUbc9 or siSUMO-1 (Figure 2C and Supplementary Figure S1C). In addition, we found that Ubc9 and SUMO-1 silencing had no effect on MAT2A mRNA level (data not shown), but MATα2 protein level fell markedly by ~65% (siUbc9) to ~80% (siSUMO-1) in HepG2 and RKO cells (Figure 2C and Supplementary Figure S1C). These results suggest that sumoylation machinery and MATα2 protein play key roles on Bcl-2 expression at both transcriptional and post-translational levels. In addition, MATα2 protein stability may also be controlled by sumoylation.

### DNA binding of MATα2 to *Bcl-2* P2 promoter sequences

**Figure 3:**
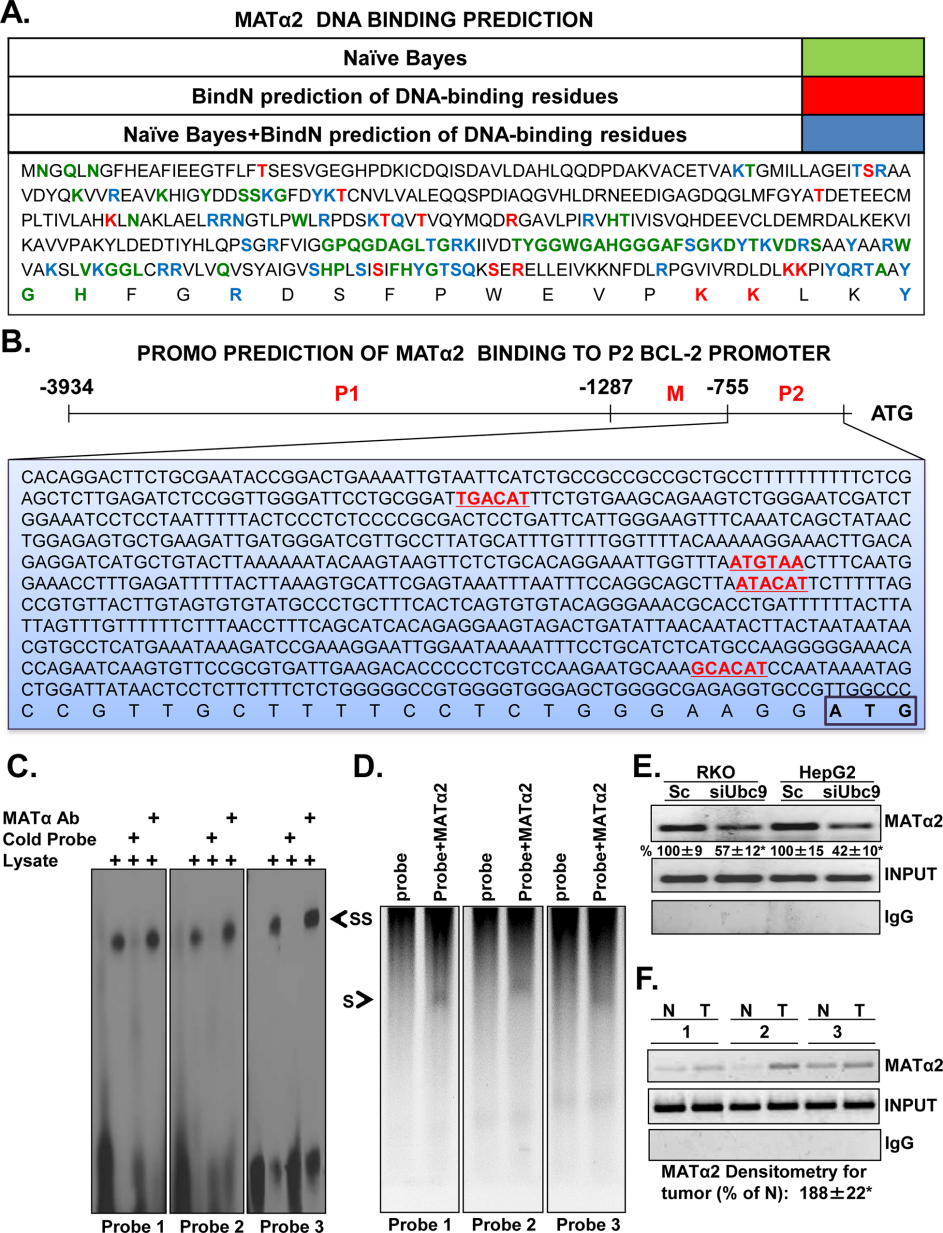
MATα2 binds to predicted *Bcl-2* P2 promoter elements **A.** Shows MATα2 protein sequences that are predicted to bind DNA by either Naïve Bayes (green), BindN (red), or both (blue) software tools. **B.** Promo software predicts *Bcl-2* P2 promoter elements that could be potentially bound by MATα2. **C.** Biotin-labeled probes specific for sites 1, 2 and 3 (designed as probes 1–3, 1 being most upstream from ATG) were incubated with nuclear protein extract from control RKO cells in the presence or absence of MATα2 antibody as described in Methods. The arrow indicates the position of the supershifted band. **D.** Shows direct binding of recombinant MATα2 to the same probes. **E.** and **F.** show MATα2 binding to the *Bcl-2* P2 promoter and siUbc9's effect using ChIP assay in RKO and HepG2 cells (E), human colon cancer specimens (T) and corresponding non–tumorous (N) tissues (F). Results are from two experiments done in duplicate for E, *p < 0.01 vs. Sc for E. Input genomic DNA was used as a positive control and IgG was used as a negative control. Densitometric changes from 5 colorectal cancers expressed as % of corresponding non-tumorous (N) tissues are summarized below blots in F, *p < 0.04 vs. non-tumorous tissue.

*Bcl-2* transcription is regulated positively by *Bcl-2* P2 promoter activity [23]. Using BindN [24] and Naïve Bayes prediction DNA-binding models, we identified several potential DNA-binding sites present in the MATα2 protein sequence (Figure 3A). Also, we identified four putative MATα2 protein binding sites on *Bcl-2* P2 promoter sequence from −749 to +1 using PROMO prediction software (Figure 3B). We analyzed predicted binding sites 1, 2 and 3 (Figure 3C and 3D) because the dissimilarity threshold (the parameter that controls how similar a sequence must be to the matrix) was higher than 15% rated by PROMO software. To critically analyze the ability of MATα2 protein to bind *Bcl-2* P2 promoter *in vitro*, gel retardation and supershift analysis of these individual elements were performed. Double-stranded oligonucleotides (Supplementary Table S1) corresponding to the putative *Bcl-2* P2 promoter MATα2 binding sites were examined for their ability to interact with RKO nuclear protein lysate. This analysis demonstrated that all three *Bcl-2* P2 promoter sites specifically interacted with MATα2, forming complexes that supershifted in the presence of anti-MATα2 antibody (Figure 3C). Same results were obtained using nuclear protein lysate from HepG2 cells (data not shown). Direct interaction between MATα2 and these DNA binding sites was further confirmed using recombinant MATα2 (Figure 3D).

### MATα2 binds to *Bcl-2* P2 promoter in HepG2, RKO cells and human colon cancer specimens

The results above show that MATα2 protein binds *Bcl-2* P2 promoter elements. We next used the chromatin immunoprecipitation (ChIP) assay to confirm that MATα2 can bind to *Bcl-2* P2 promoter region in endogenous chromatin configuration in living cells and examine whether this is increased in human colon cancer where MATα2 is overexpressed [20]. MATα2 strongly interacts with the *Bcl-2* P2 promoter and Ubc9 knockdown lowered this binding in both RKO and HepG2 cells by 43% and 58%, respectively (Figure 3E). Figure 3F shows that MATα2 exhibited enhanced binding to the *Bcl-2* P2 promoter region in colorectal cancer compared with corresponding surrounding non-tumorous tissues by 88%.

### MATα2 sumoylation *in vitro*

**Figure 4:**
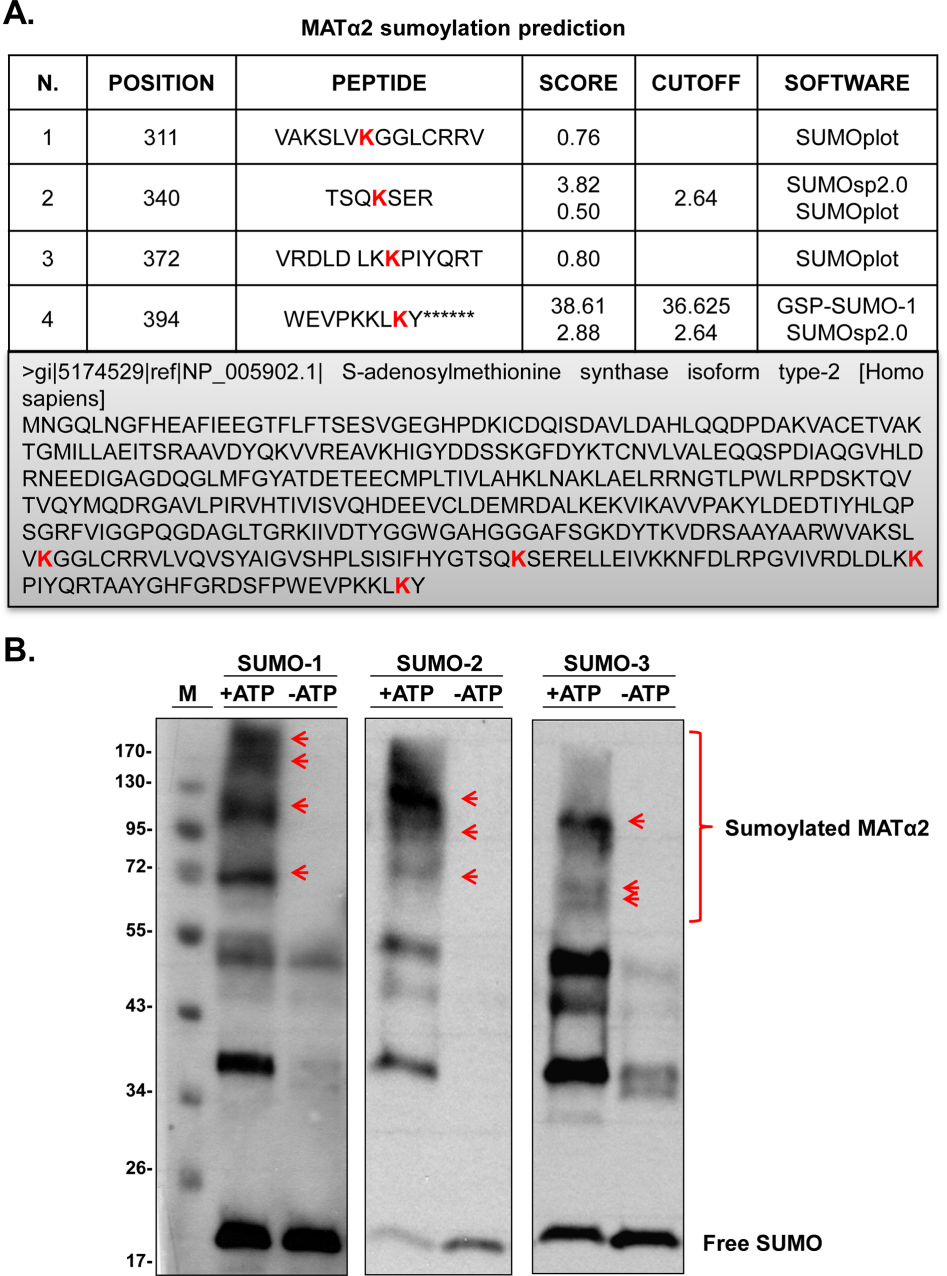
SUMO-binding prediction of MATα2 and *in vitro* sumoylation **A.** Prediction of potential sumoylated sites in MATα2 protein sequence using the SUMOsp2.0, SUMOplot and GSP-SUMO-1 analysis tools. **B.** shows sumoylation of MATα2 by SUMO-1, SUMO-2 and SUMO-3 recombinant protein system as described in Methods. Sumoylation was measured using anti-SUMO-1 and anti-SUMO-2/3 antibodies in Western blotting. Red arrows indicated sumoylated MATα2.

Our results show that Ubc9 and SUMO-1 knockdown lower MATα2 protein level in both HepG2 and RKO cells (Figure 2C and Supplementary Figure S1C). Sumoylation by SUMO-1 has been shown to control stability of its protein target [25]. Using SUMOplot, SUMOsp2.0 and GSP-SUMO-1 sumoylation prediction software, we identified four potential sumoylated sites on MATα2 protein sequence (Figure 4A). To investigate whether MATα2 is sumoylated *in vitro*, we carried out *in vitro* sumoylation assays using highly purified MATα2 recombinant protein and commercially available SUMOylation assay kit [26]. Sumoylated target proteins show higher molecular weight compared with wild-type (WT) proteins [27]. Figure 4B shows that MATα2 is sumoylated by SUMO-1, SUMO-2 and SUMO-3.

### MATα2 is sumoylated in human colorectal tissues, HepG2 and RKO cells

**Figure 5:**
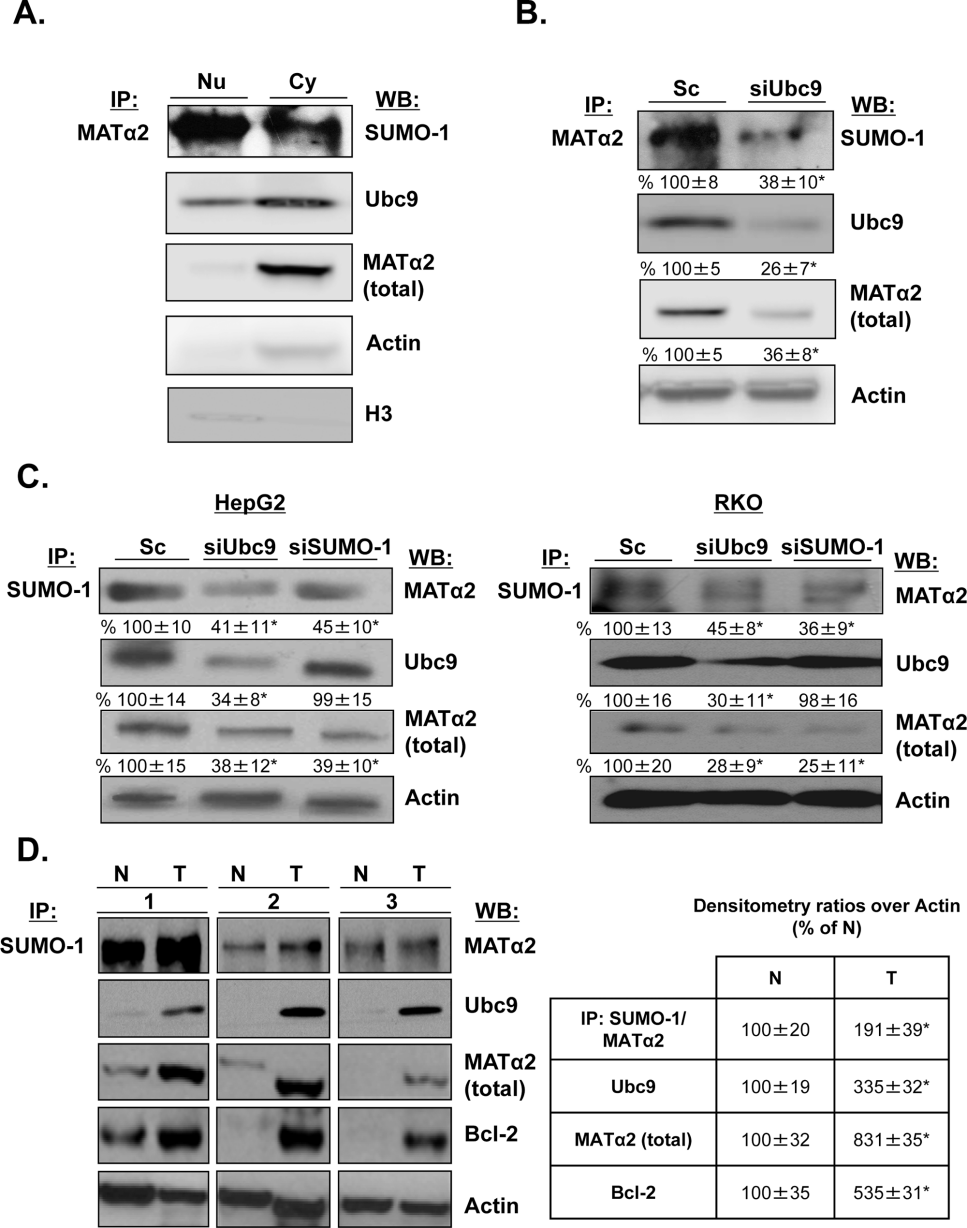
MATα2 is sumoylated in HepG2 and RKO cells and human colon cancers **A.** Nuclear and cytoplasmic proteins from HepG2 cells were immunoprecipitated with anti-MATα2 and Western blotting was carried out with anti-SUMO-1, MATα2 and Ubc9 antibodies. **B.** and **C.** HepG2 and RKO cells were treated with siUbc9 and siSUMO-1 or scrambled (Sc, 20 nM) for 48 hours and co-immunoprecipitation of MATα2 or SUMO-1 was performed followed by Western blotting with anti-MATα2 and anti-Ubc9 antibodies. Densitometric values are shown below the blots, and results represent mean ± SEM from 3 experiments done in duplicates expressed as % of Sc, *p < 0.04 *vs.* Sc. **D.** Sumoylation of MATα2 in human colon cancer specimens (T) and corresponding non–tumorous (N) tissues are shown. The immunoprecipitation was done as described above for sumoylated MATα2; total MATα2, Ubc9 and Bcl-2 are also measured in the same specimens using Western blotting. Densitometric changes are shown in adjacent box, and results represent mean ± SEM from 5 colorectal cancers expressed as % of corresponding non-tumorous (N) tissues, *p < 0.05 vs. non-tumorous tissue.

Since MATα2 is localized in both nuclear and cytoplasmic compartments [28], we investigated whether MATα2 is sumoylated *in vivo* and where the sumoylated form is localized in HepG2 cells. Figure 5A shows that MATα2 is sumoylated in both compartments by SUMO-1; however, despite the fact that the bulk of total MATα2 is in the cytoplasmic compartment, there is much more sumoylated MATα2 in the nucleus. Knocking down Ubc9 reduced both SUMO-1-sumoylated and total MATα2 (Figure 5B). Reverse IP using anti-SUMO-1 antibody followed by Western blotting confirmed MATα2 sumoylation by SUMO-1, which is reduced comparably by either Ubc9 or SUMO-1 knockdown in HepG2 and RKO cells (Figure 5C). Finally, Figure 5D shows in paired colon cancer and adjacent non-tumorous tissues, MATα2 is SUMO-1 sumoylated and SUMO-1-MATα2 level is nearly doubled in colon cancer. In the same specimens the protein levels of Ubc9, MATα2 and Bcl-2 are all higher in cancer.

### Bcl-2 directly interacts with MATα2 and Ubc9 in HepG2 and RKO cells

**Figure 6:**
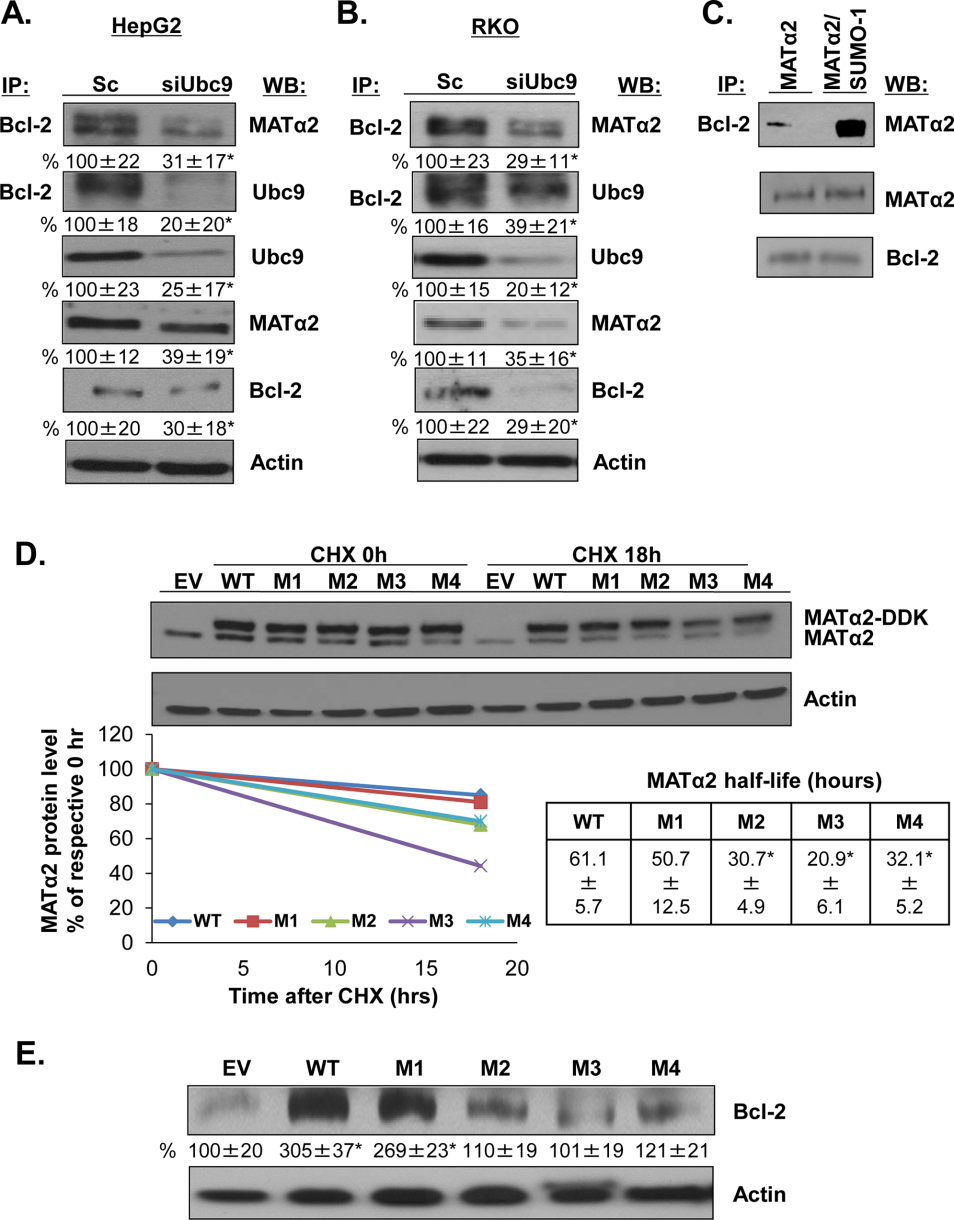
MATα2 directly interacts with Bcl-2 and mutation at MATα2 SUMO-binding sites affects MATα2 stability and Bcl-2 protein level **A.** and **B.** HepG2 and RKO cells were transfected with scrambled (Sc) or siUbc9 (20 nM) for 48 hours. Co-IP analysis and Western blotting were done as described in Methods. Results are expressed as % of Sc (mean ± SEM) from 4 independent experiments and shown below the blots, *p < 0.04 vs. Sc. **C.** Recombinant MATα2 and Bcl-2 proteins interaction *in vitro* was done as described in Methods. **D.** RKO cells were transfected with empty vector (EV), hMAT2A-WT, hMAT2A-M1, hMAT2A-M2, hMAT2A-M3 or hMAT2A-M4 for 24 hours as described in Methods. MATα2 protein stability was determined by Western blot analyses of DDK tag following cycloheximide (CHX) treatment as described in Methods. Representative blots are shown. Protein stability was determined using linear regression and half-life calculated using equation indicated. Results represent mean ± SEM from 3 independent experiments expressed as % of respective 0 hr level (*p* < 0.04 between WT and MUT). **E.** Effects of overexpressing hMATα2-WT or MATα2-mutants for 36 hours on Bcl-2 protein level. Densitometric results are expressed as % EV control (mean ± SEM) from 3 independent experiments and shown below the blot. *p < 0.01 vs. EV.

Since MATα2 and Ubc9 knockdown lowered Bcl-2 protein level further than the mRNA level, we examined whether Bcl-2 interacts with MATα2 and Ubc9 in HepG2 and RKO cells. Figure 6A and 6B show that Bcl-2 co-immunoprecipitated with MATα2 and Ubc9 and silencing of Ubc9 lowered their interaction. Also, we tested whether MATα2 and Bcl-2 physically interact *in vitro* using highly purified Bcl-2 and MATα2 recombinant proteins. Figure 6C shows that MATα2 and Bcl-2 form a complex *in vitro* and sumoylated MATα2 leads to higher complex formation.

### Mutation of MATα2 sumoylation binding sites influence protein stability of MATα2 and Bcl-2

Since knockdown of either Ubc9 or SUMO-1 lowered MATα2 protein level without affecting the mRNA level, we hypothesize that sumoylation regulates MATα2 protein stability. We examined whether the predicted SUMO binding sites (Figure 4A) are relevant for MATα2 protein stability by mutating K311, K340, K372 and K394 and measured MATα2 protein stability in RKO cells. For this purpose, we created mutations in the overexpression vector of hMAT2A DDK-tagged at K311, K340, K372 and K394 (to R311, R340, R372 and R394) (Supplementary Table S2) and examined the total MATα2 protein level using Western blotting analysis. Figure 6D shows that mutation at K340 (M2), K372 (M3) and K394 (M4) (but not K311, or M1) resulted in a less stable MATα2 as demonstrated by measurement of protein half-life in RKO cells. Overexpressing mutant MATα2 constructs had no influence on the endogenous MATα2 protein stability (results not shown). To test whether MATα2 is required for Bcl-2 protein stability, we tested the effect of expressing MATα2 WT or mutants for 36 hours on Bcl-2 protein level by Western blotting analysis in RKO cells. Figure 6E shows that MATα2 WT and M1 overexpression increased Bcl-2 protein level by about 2-fold compared to empty vector control, whereas mutants M2, M3 and M4 overexpression had no effect on Bcl-2 protein level. Overexpression of wt and mutant MAT2A constructs was able to raise MAT2A mRNA level more than 300 fold over empty vector at 24 hours (Supplementary Figure S2A). Overexpressing WT, M1 and M4 also raised Bcl-2 mRNA level comparably, whereas overexpressing M2 and M3 did not significantly affect Bcl-2 mRNA level (Supplementary Figure S2B).

### Overexpressing stable forms of MATα2 protects cancer cells from 5-fluorouracil (5-FU)-induced apoptosis

**Figure 7:**
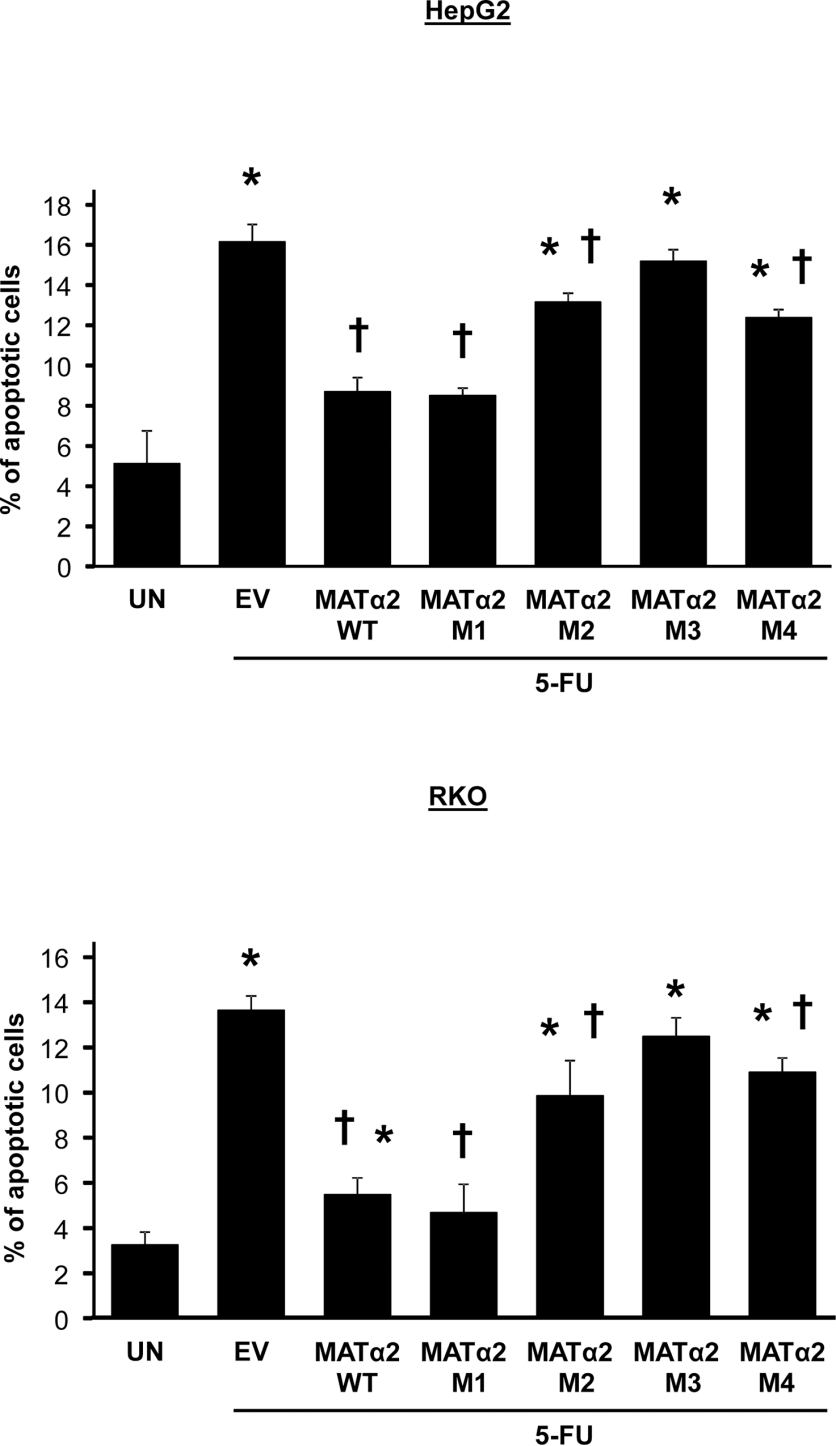
Overexpression of wild type but not sumoylation mutants that result in less-stable MATα2 protect against 5-FU-induced apoptosis HepG2 and RKO cells were transfected with empty vector (EV), hMAT2A-WT, hMAT2A-M1, hMAT2A-M2, hMAT2A-M3 or hMAT2A-M4 for 24 hours followed by treatment with 5-FU as described in Methods. Results are expressed as % apoptotic cells from 3 independent experiments, *p < 0.04 vs. untreated (UN), †*p* < 0.04 vs. EV.

Overexpressing WT and M1 sumoylation MATα2 mutant (stability not affected) protected against 5-FU-induced apoptosis in both HepG2 and RKO cells but M2-M4 sumoylation MATα2 mutants were either ineffective or minimally effective (Figure 7).

### Effects of MATα2 on Bcl-2 expression are independent of SAMe

**Figure 8:**
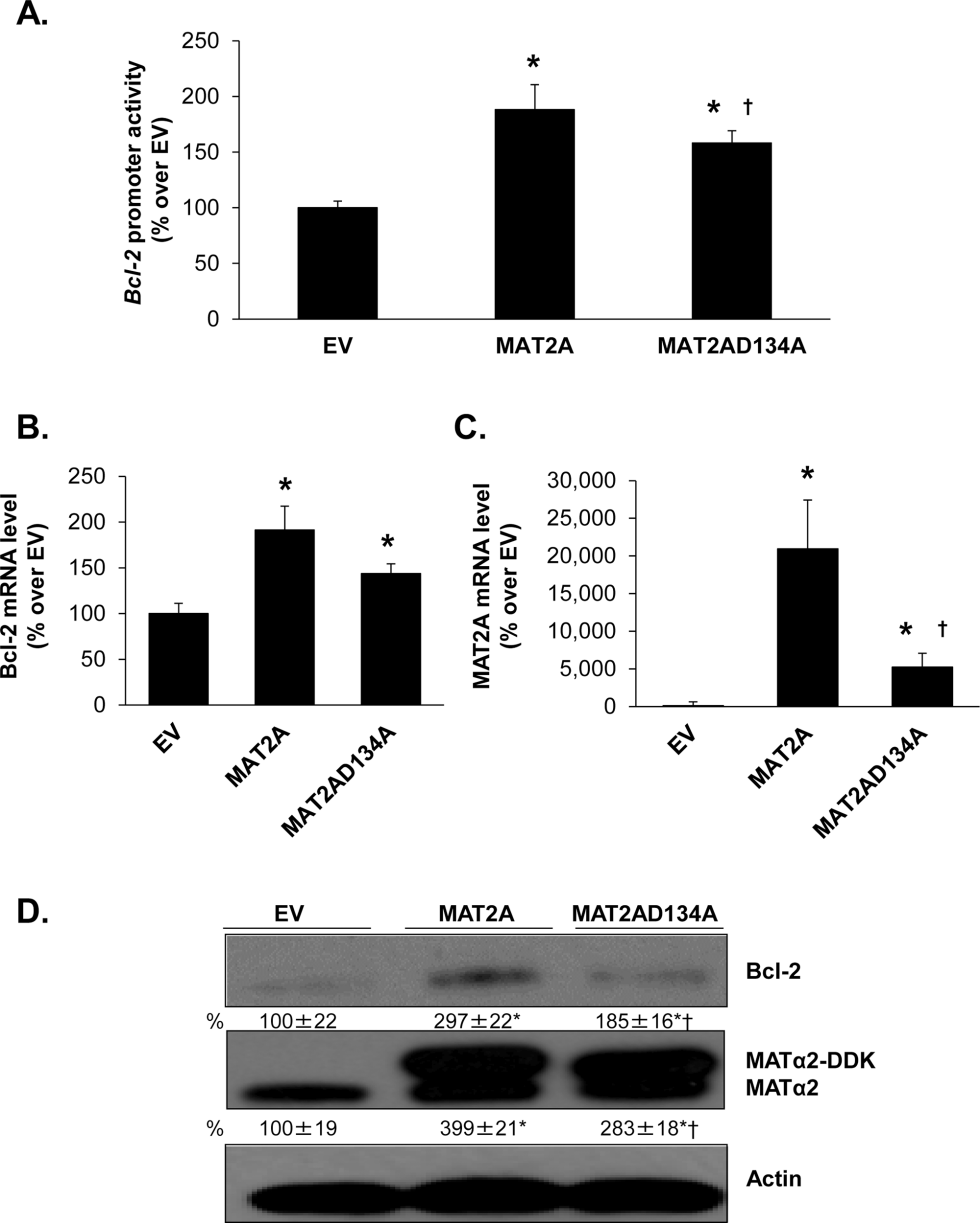
Effect of overexpressing MATα2 catalytic mutant on Bcl-2 promoter activity and expression RKO cells were transfected with wild type (WT) or catalytic mutant MAT2A overexpression vector or empty vector (EV) for 24 hours, with or without the human Bcl-2 promoter construct as described in Methods. **A.** shows effect of overexpressing the MAT2A catalytic mutant as compared to WT on *Bcl-2* promoter activity, expressed as % over EV. Results represent mean ± SEM from 5 experiments in duplicate, *p < 0.0001 vs. EV control; †*p* < 0.05 vs. WT MAT2A. **B.** and **C.** show effect of overexpressing WT or mutant MAT2A on Bcl-2 and MAT2A mRNA levels. Results represent mean ± SEM from 4 experiments in duplicates, *p < 0.002 vs. EV control; †*p* < 0.05 vs. WT MAT2A. **D.** Cells were treated as in ‘A’ and total cellular protein was subjected to Western blotting with antibodies against MATα2 or Bcl-2. Densitometric changes were normalized to actin. Representative images and densitometric analysis (% mean ± SEM of EV, indicated below the blots) from 6 experiments are shown. *p < 0.05 vs. EV, †*p* < 0.05 vs. WT MAT2A overexpression vector.

Since MATα2 catalyzes the formation of SAMe, which could affect gene and protein expression indirectly, we compared overexpression of wild type MATα2 or MATα2 catalytic mutant (MATα2 D134A) which lacks catalytic function [28]. Overexpression of either wild type or catalytic mutant of MATα2 raised Bcl-2 promoter activity (Figure 8A), mRNA (Figure 8B) and protein levels (Figure 8D), albeit the wild type construct was more inductive. This may be related to lower expression of the mutant MATα2 (Figures 8C and 8D).

## DISCUSSION

Sumoylation modifies the activity of key regulatory proteins, including oncoproteins, tumor suppressors, cell cycle regulators, and enzymes involved in DNA repair [29]. Therefore, alterations in protein sumoylation and de-sumoylation caused by deregulation of Ubc9 alone or any component of the sumoylation machinery would affect the cellular pathways linked to tumorigenesis and related biological consequences [30, 31]. Sumoylation modulates many proteins implicated in apoptosis such as Fas, TNFR1, Daxx, p53 and its regulator MDM2 [32, 33] and Ubc9 has been demonstrated to regulate *Bcl-2* expression through the estrogen receptor signaling pathway in MCF-7 cells [13].

Our earlier works led us to suspect there may be interplay between sumoylation, MAT2A and Bcl-2. Specifically, Ubc9 and MAT2A expression are increased in both liver and colon cancers and SAMe, which lower both Ubc9 and MAT2A expression, induces apoptosis in these cancer cells [16]. Bcl-2 is an oncoprotein that function by promoting cancer cell survival rather than proliferation [34]. Increased Bcl-2 expression is linked to chemoresistance [35] and targeting this has been an emerging strategy in cancer treatment [36]. To examine interaction between sumoylation, MAT2A and Bcl-2, we used HepG2 and RKO cells as SAMe treatment lowered MAT2A and Ubc9 expression and induced apoptosis in these cells [10, 17, 20]. Moreover, both cell types do not express estrogen receptor [37, 38]. We found that similar to MCF-7 cells [13], knockdown of Ubc9 in HepG2 and RKO cells also lowered Bcl-2 expression. However, the mechanism is independent of the estrogen receptor. Of considerable interest, Ubc9 knockdown lowered Bcl-2 protein level much more than mRNA level (Figure 1C–1D and Supplementary Figure S1C–S1D), suggesting that Ubc9 affects Bcl-2 expression at both transcriptional and post-translation levels. Ubc9 knockdown induced apoptosis, similar to SAMe and MTA treatment in HepG2 and RKO cells (Figure 1A–1B). SAMe and MTA treatment lowered Bcl-2 expression (Figure 1E–1F), an effect that has not been previously reported and likely also contribute to their pro-apoptotic effect in these cancer cell lines.

SAMe is the principal methyl donor and precursor of polyamines synthesized by *MAT1A* and *MAT2A*-encoded isoenzymes [16]. *MAT1A* is often silenced in human liver cancer and overexpression of *MAT1A* in liver cancer cells resulted in higher SAMe level and increased apoptosis [39]. Our current results are consistent with the notion that higher SAMe level led to reduced Bcl-2 expression observed in MAT1A transfected HepG2 cells [40]. MAT2A expression is increased in human colon cancer tissues and cells treated with mitogens, whereas silencing MAT2A resulted in apoptosis [19, 20]. In both HepG2 and RKO cells, knockdown of MAT2A decreased, whereas MAT2A overexpression increased Bcl-2 expression (Figure 2 and Supplementary Figure S1). This appears to contradict the effect of exogenous SAMe treatment since MAT2A knockdown reduced intracellular SAMe level [17]. Thus, the effect of MAT2A expression on Bcl-2 appears to be independent of SAMe and is very similar to the effect of Ubc9 so that both MAT2A and Ubc9 knockdown lowered Bcl-2 expression at the promoter level as well at the protein level. Furthermore, overexpression of MAT2A increased *Bcl-2* promoter activity and protein level but this effect required Ubc9 (Figure 2 and Supplementary Figure S1). Since Ubd9 can have sumoylation-dependent and independent actions [41], we also examined the effect of SUMO-1 knockdown on Bcl-2 expression, which produced essentially the same effect as Ubc9 and MAT2A knockdown. Importantly, both SUMO-1 and Ubc9 knockdown drastically reduced MATα2 protein level, without affecting MAT2A mRNA level. Taken together, these results support our conclusion that MATα2 is sumoylated by SUMO-1, which stabilizes MATα2 protein and MATα2 is the link between Ubc9 and Bcl-2 expression in these cells.

MATα2 regulates Bcl-2 expression at both the promoter and protein levels. This is supported by the fact that the effect is more pronounced at the protein level when MAT2A is knocked down (Figure 2 and Supplementary Figure S1). In the only report thus far on MATα2's ability to regulate at the transcriptional level, MATα2 was found to be a transcriptional corepressor by providing a local source of SAMe and interacting with chromatin-related factors [28]. However, our results suggest MATα2 can act as a bona fide transcription factor as overexpression of the catalytic mutant was equally effective in raising Bcl-2 promoter activity (Figure 8). The ability of MATα2 to bind DNA is unknown and it has not been shown to activate genes transcriptionally. Consistent with previous report on transcriptional factors DNA binding [42], we found that MATα2 protein could potentially bind to DNA by its C-terminal domain (Figure 3A). Following the prediction data in Figure 3B, we next examined the binding ability of MATα2 to three potential *Bcl-2* P2 promoter binding sites (Figure 3B and Supplementary Table S1). We focused on the P2 promoter because the P2 promoter positively regulates Bcl-2 expression [23]. We confirmed that MATα2 can bind to all three predicted binding sites on electrophoretic mobility shift assay (EMSA) and supershift assays and the interaction is direct and requires no other proteins (Figure 3C–3D). Furthermore, these findings were verified by ChIP assays, which clearly demonstrated that MATα2 interacts with *Bcl-2* P2 promoter in cells and in human colon cancer samples (Figure 3F). MATα2 binding to the *Bcl-2* P2 promoter was reduced by Ubc9 knockdown, which is likely due to the fact that MATα2 protein level was reduced. However, we cannot rule out the possibility that sumoylation also influences protein-protein interaction and/or MATα2's ability to bind DNA as sumoylation can affect DNA-binding activity of transcription factors [43].

Given the fact that both SUMO-1 and Ubc9 knockdown drastically reduced MATα2 protein level without affecting its mRNA level, we suspect critical sumoylation sites are present in the MATα2 protein sequence that affect its stability. Consistently, four potential sites are present in its protein sequence and we demonstrate MATα2 can be sumoylated *in vitro* by SUMO-1 as well as SUMO-2/3 (Figure 4B). We focused on SUMO-1 because it is known to stabilize proteins [3], while SUMO-2/3 chain formation promotes targeted protein degradation via proteasome [5]. We confirmed *in vivo* that MATα2 is sumoylated by SUMO-1 at baseline and Ubc9 and SUMO-1 silencing resulted in decreased MATα2 protein level (Figure 5B–5C). Interestingly, SUMO-1 sumoylated MATα2 is preferentially localized in the nuclear compartment (Figure 5A), and SUMO-1 sumoylation has been shown to play a key role for nuclear targeting and function of transcription factor ZIC3 [44]. We speculate a similar role may be true for SUMO-1 sumoylation of MATα2, which will require further examination. MATα2 is also SUMO-1 sumoylated in normal colon tissue and this is enhanced in colon cancer and higher protein levels of Ubc9, MATα2 and Bcl-2 all occur in the cancer tissues (Figure 5D). Through point mutation analysis and cycloheximide (CHX) pulse-chase experiments, we determined that the critical sites for MATα2 stability were at the K340, K372, and K394 found in the C-terminus (Figure 6D and Supplementry Table S2). Therefore, SUMOylation of MATα2 plays a critical role in its protein stability and this is likely achieved by SUMO-1. The functional outcome(s) of SUMO-2/3 sumoylation of MATα2 is unclear and will be a subject of future investigation.

MATα2 also regulates Bcl-2 at the protein level. To our surprise, we found that MATα2 physically interacts with Bcl-2 directly and sumoylated MATα2 formed complex with Bcl-2 more efficiently *in vitro* (Figure 6C). Consistently, we found that Ubc9 knockdown resulted in decreased Bcl-2/MATα2 complex in HepG2 and RKO cells (Figure 6A–6B). It is noteworthy that overexpression of MATα2 sumoylation mutants M2, M3 and M4 was unable to raise Bcl-2 protein level (Figure 6E), suggesting that Bcl-2/MATα2 complex formation requires MATα2 sumoylation and may protect Bcl-2 from degradation. This is supported by the finding that despite comparable elevation in Bcl-2 mRNA levels by WT, M1 and M4 MAT2A constructs, only WT and M1 raised Bcl-2 protein level. However, sumoylation at K340 (M2) and K372 (M3) may also be important for MATα2 to bind and activate the *Bcl-2* promoter since Bcl-2 mRNA level did not increase with their overexpression. This is also consistent with the result that Ubc9 knockdown lowered MATα2 binding to the *Bcl-2* promoter (Figure 3D). Finally, we demonstrated the significance of MATα2 overexpression on conferring chemoresistance as protection against 5-FU-induced apoptosis was most pronounced in cancer cells overexpressing wild type or sumoylation mutant that does not affect MATα2 stability (Figure 7).

**Figure 9:**
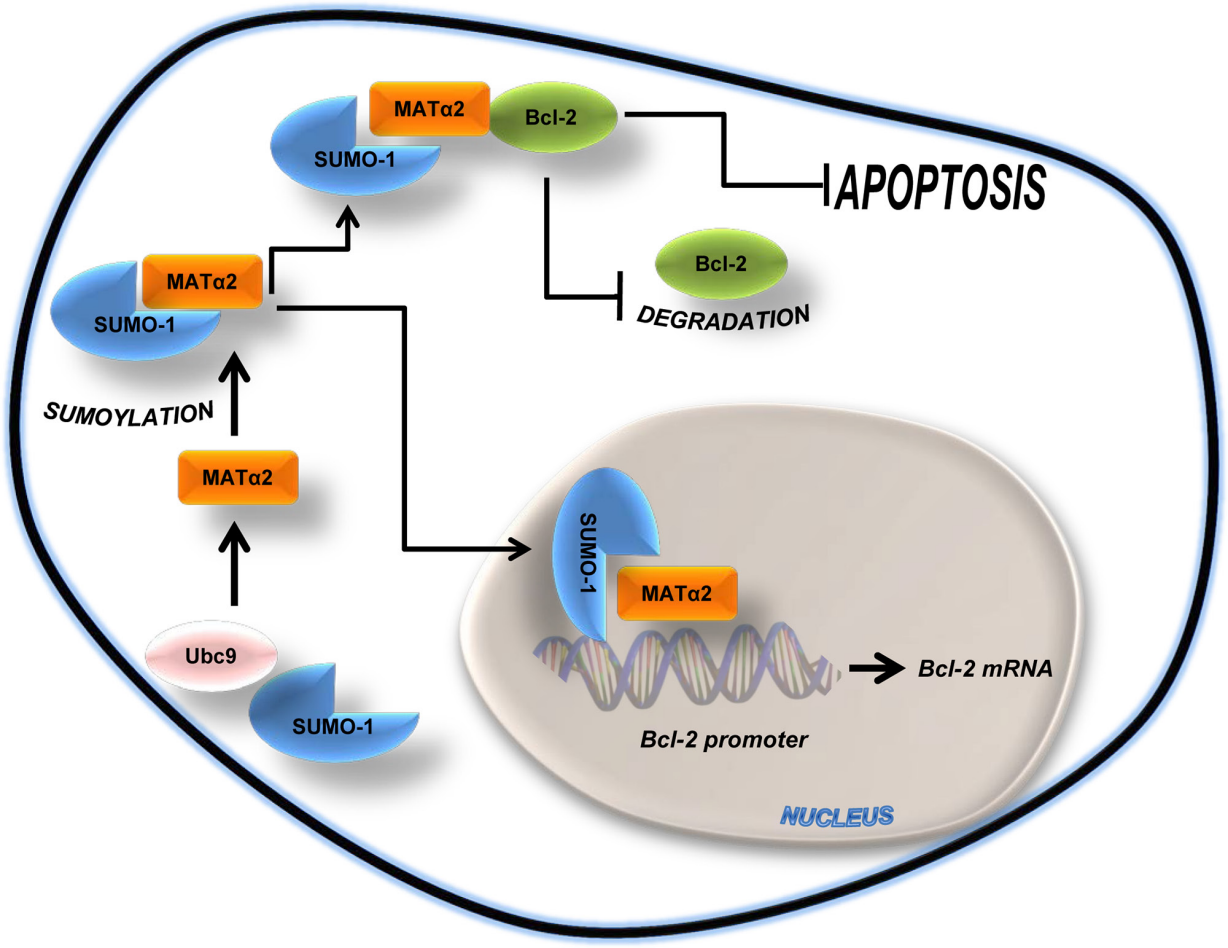
Simplified schematic of key findings MATα2 is sumoylated by SUMO-1 at basal level. Sumoylated MATα2 regulates *Bcl-2* mRNA level via binding to *Bcl-2* P2 promoter elements and increasing its activity. Sumoylated MATα2 also forms a protein complex with Bcl-2, resulting in higher Bcl-2 protein stability. This positive feed forward loop protects against apoptosis.

In summary, our current work uncovered, to our knowledge, a previously unreported function of MATα2, namely the ability to act as a transcriptional factor that positively regulates the transcriptional activity of *Bcl-2*. In addition, MATα2 is sumoylated, which stabilizes the protein. Sumoylated MATα2 is able to interact better with Bcl-2 and this interaction stabilizes Bcl-2. These results are summarized in Figure 9. Our findings show MATα2 regulates Bcl-2 expression at transcriptional and post-translational levels and is the dominant link between Ubc9 and Bcl-2. These findings provide novel insights on MATα2's transcriptional factor function, regulation of Bcl-2, and how the interplay between Ubc9, MATα2 and Bcl-2 can confer survival benefit to the cancer cell.

## MATERIALS AND METHODS

### Materials

SAMe, in the stable form of disulfate p-toluene sulfonate dried powder was generously provided by Gnosis SRL (Cairate, Italy) and MTA, CHX, 5-FU, and SYBR^®^ Green I nucleic acid gel staining were from Sigma-Aldrich (St. Louis, MO). MATα2 and Bcl-2 recombinant proteins were purchased from GenWay Biotech (San Diego, CA). All other reagents were of analytical grade and obtained from commercial sources.

### Cell culture and SAMe/MTA treatment

HepG2 and RKO cells were obtained from the Cell Separation and Culture Core of the University of Southern California Liver Disease Research Center and grown according to instructions provided by American Type Cell Collection. Cells were grown in 10% fetal bovine serum for 24 hours and media was changed prior to SAMe (2 mmol/L) or MTA (1 mmol/L) treatment for another 24 hours. Because of the instability of SAMe, medium was changed every 6 hours for all conditions in experiments that included SAMe.

### Tissue specimens

Five pairs of colon cancer and corresponding surrounding non-tumorous tissue from surgical resection for primary colon cancer were used. Colon tissues were obtained by Professor Giordano P (Whipps Cross University Hospital, London, UK). These tissues were immediately frozen in liquid nitrogen for subsequent protein extraction. Written informed consent was obtained from each patient. The study protocol conformed to the ethical guidelines of the 1975 Declaration of Helsinki as reflected in a priori approval by Keck School of Medicine University of Southern California's human research review committee.

### RNA extraction and real-time polymerase chain reaction analysis

Total RNA isolated from HepG2 and RKO cells and colon tissues as described [45] was subjected to reverse transcription (RT) by using M-MLV Reverse Transcriptase (Invitrogen, Carlsbad, CA). One μl of RT product was subjected to quantitative real-time PCR analysis. The primers and TaqMan probes for Ubc9, Bcl-2 and MAT2A and Universal PCR Master Mix were purchased from ABI (Foster City, CA). 18SrRNA was used as housekeeping gene as described [20]. The delta Ct (ΔCt) obtained was used to find the relative expression of genes according to the formula: relative expression *n* = 2^−ΔΔCt^, where ΔΔCt= ΔCt of respective genes in experimental groups–ΔCt of the same genes in control group.

### RNA interference

The predesigned siRNA targeting human MAT2A (sense sequence: ACACAUUGGAUAUGAUGAUTT) was purchased from Invitrogen (Carlsbad, CA), while Ubc9 (sense sequence: GGAACUUCUAAAUGAAC CATT), SUMO-1 (sense sequence: AAGUGAAUAU AUUAAACUCA), and scrambled control siRNA were purchased from Ambion (Austin, TX). RKO and HepG2 cells were cultured in 6-well plate (0.4×10^6^ cells/well) and transfected using RNAiMax (5 μL/well) from Invitrogen (Carlsbad, CA) with MAT2A, Ubc9 and SUMO-1 siRNA (20 nM), or scrambled control siRNA for 48 hours for mRNA or protein expression, following the manufacturer's instructions.

### Western blots and co-immunoprecipitation (co-IP)

Total protein extracts from HepG2, RKO and colon samples were prepared as described [17] while nuclear and cytoplasmic proteins from HepG2 were prepared using EpiQuick Nuclear Extraction Kit I (Epigenetek, Farmingdale, NY). Protein extracts were immunoprecipitated by specific MATα2, SUMO-1 or Bcl-2 antibodies and processes as reported [46]. Immunoprecipitated proteins were subjected to Western blotting following standard protocols (Amersham BioSciences, Piscataway, NJ), and the membranes were probed with the anti-MATα2 (Novus, Littleton, CO), anti-SUMO-1, anti-Ubc9 (Genetex, Irvine, CA) and anti-Bcl-2 (Santa Cruz Biotechnology, Santa Cruz, CA) antibodies. Blots were developed using enhanced chemoluminescence.

### Overexpression of wt and mutant MAT2A

Myc-DDK-tagged MAT2A overexpression vector (MAT2A-pCMV6) and negative control empty vector (pCMV6) were purchased from Origene (Rockville, MD). Catalytic MATα2 mutant (D134A) lacking catalytic function was prepared as previously described [28] and verified by sequencing. Mutations of MATα2 SUMO-binding Lysine sites (K311, K340, K372, K394) to Arginine were performed by Quick Change II Site-Directed Mutagenesis Kit (Stratagene, La Jolla, CA) with pCMV6 as the template. All of the primers were synthesized by Valuegene (San Diego, CA) and simply PAGE purified (Supplementary Table S2). RKO and HepG2 cells were cultured in 6-well plates (0.4×10^6^ cells/well), transfected using 5 μl of JetPRIME from Polyplus (New York, NY) and 2 μg of target plasmid per well. After 4 hours, the transfection medium was changed to normal medium and the cells were cultured for an additional 20 hours for promoter activity assay, mRNA and protein expression as indicated.

### *Bcl-2* promoter reporter assay

The *Bcl-2* promoter-luciferase reporter plasmid pBS (Addgene, Cambridge MA), WT and catalytic mutant MATα2 expression plasmids were transfected into HepG2 and RKO cells (0.4×10^6^ cells/well, 6-well plates) using the BioT reagent (Bioland Scientific, Paramount, CA). As an internal control, the pRL4.73 plasmid (Promega, Madison, WI), which carries a Renilla luciferase gene, was co-transfected into the cells. The cells were lysed to measure both firefly and Renilla luciferase activities by the Dual-Luciferase Activity Detection System (Promega) 24 hours post transfection. Relative luciferase activity was calculated by normalizing the ratio of firefly/Renilla luciferase activity to that of mock-transfected cells. In some experiments cells were first treated with Ubc9, MAT2A or SUMO-1 siRNA for 24 hours prior to co-transfection with *Bcl-2* promoter construct and MATα2 expression plasmid.

### EMSA and supershift assay

Nuclear extracts were prepared according to the EpiQuik™ Nuclear Extraction Kit I protocol (Epigentek, Farmingdale, NY). Extracts were subjected to EMSA and supershift (3 μg MATα2 antibody) using the LightShift^®^ Chemiluminescent EMSA Kit protocol (Thermo Scientific, Waltham, MA) and probes as described (Supplementary Table S1). In other experiments, recombinant MATα2 (200 ng) was used for EMSA instead of nuclear extracts and nucleic acids are detected with SYBR^®^ Green dye (1:10,000) incubating the gels for 20 min at room temperature, and rinsed twice with deionized H2O. The gels were visualized using UVP ChemiDoc-It^t32^ Imager (UVP, Upland, CA).

### ChIP assay

ChIP assays were performed using the *EZ*-ChIP kit (Millipore, Billerica, MA). Sonicated chromatin immunoprecipitated with 5 μg of antibody against MATα2 was reverse cross-linked and PCR amplified for 35 cycles with the following Forward (Fw) and Reverse (Rev) *Bcl-2* P2 promoter primer sequences: Fw, 5′-GATTCCTGCGGATTGACATTTCT-3′; Rev, 5′-CCAAGAATGCAAAGCACATCCAA-3′.

### *In vitro* sumoylation of MATα2

Recombinant MATα2 (1 μg) was sumoylated *in vitro* using SUMOylation kit (Enzo Life Sciences, Farmingdale, NY) in a final volume of 10 μl containing 55 mM Tris (pH 7.5), 5.5 mM MgCl_2_, 2.2 mM ATP and 5.5 mM dithiothreitol. Sumoylation reactions were incubated at 37°C for 4 hours, after which proteins were analyzed by Western blotting with SUMO-1 and SUMO-2/3 antibodies.

### MATα2 and Bcl-2 *in vitro* interaction assay

Recombinant Bcl-2 (1 μg) was pre-incubated with Bcl-2 agarose-conjugated antibody (20 μg) (Santa Cruz Biotechnology, Santa Cruz, CA) in 500 μL final volume of incubation buffer (150 mM NaCl, 50 mM Tris-HCl, 1 mM EDTA, 1 mM EGTA, 25 mM NaF, 1% NP-40) at 4°C on rotating device for 1 hour. Next, we added 1 μg of recombinant MATα2 or sumoylated MATα2 prepared as described above, at 4°C for 2 hours with rotation. Pellet agarose was washed five times with incubation buffer. Finally, Bcl-2/MATα2, Bcl-2/sumoylated-MATα2 complex interactions, Bcl-2 and MATα2 loading control samples (prior to *in vitro* sumoylation and complex interaction) were analyzed by Western blotting using Bcl-2 and MATα2 antibodies.

### Protein stability assay and half-life determination

CHX (5 μg/ml) was added to RKO cells 18 hours after transfection with 2 μg of wild type MATα2-pCMV4 or MATα2 sumoylation mutant plasmids per well. Protein levels were determined at indicated time points by Western blotting as described above using anti-MATα2 antibody. The relative amount of MATα2-DDK protein was evaluated by densitometry and normalized to actin. Endogenous and DDK-tagged MATα2 bands were separately analyzed. Protein half-life was determined for each experiment using linear regression analysis as we described [10].

### 5-FU treatment *in vitro* and apoptosis assay

Seventy percent confluent RKO and HepG2 cells were treated with 12.5 μM of 5-FU in fresh growth medium for 24 hours after transfection with 2 μg of wild type MATα2-pCMV4 or MATα2 sumoylation mutant plasmids per well. The percentage of apoptotic cells was examined using Hoechst 33258 staining method as we described [19].

### Statistical analysis

Data are expressed as mean ± SEM. Statistical analysis was performed using ANOVA and Fisher's test. For mRNA and protein levels, ratios of genes and proteins to respective housekeeping densitometric values were compared. Significance was defined by *p* < 0.05.

## SUPPLEMENTARY FIGURES AND TABLES



## Abbreviations

5-FU: 5-fluorouracil
Bcl-2: B-Cell CLL/lymphoma 2
ChIP: Chromatin Immunoprecipitation
CHX: cycloheximide
Co-IP: co-immunoprecipitation
EMSA: Electrophoretic mobility shift assay
MAT: methionine adenosyltransferase
MTA: methylthioadenosine
PCR: polymerase chain reaction
SAMe: S-adenosylmethionine
siRNA: small interfering RNA
SUMO: ubiquitin-related modifier
Ubc9: ubiquitin-conjugating enzyme 9
WB: Western Blotting
WT: wild type
