# Supplementary material for: Methionine adenosyltransferase α2 sumoylation positively regulate Bcl-2 expression in human colon and liver cancer cells
Source: Oncotarget. 2015 Sep 25;6(35):37706–23. doi: 10.18632/oncotarget.5342 (PMC4741959; doi:10.18632/oncotarget.5342)
Supplement: Supplementary file 1 [file oncotarget-06-37706-s001.pdf]

SUPPLEMENTARY FIGURES AND TABLES

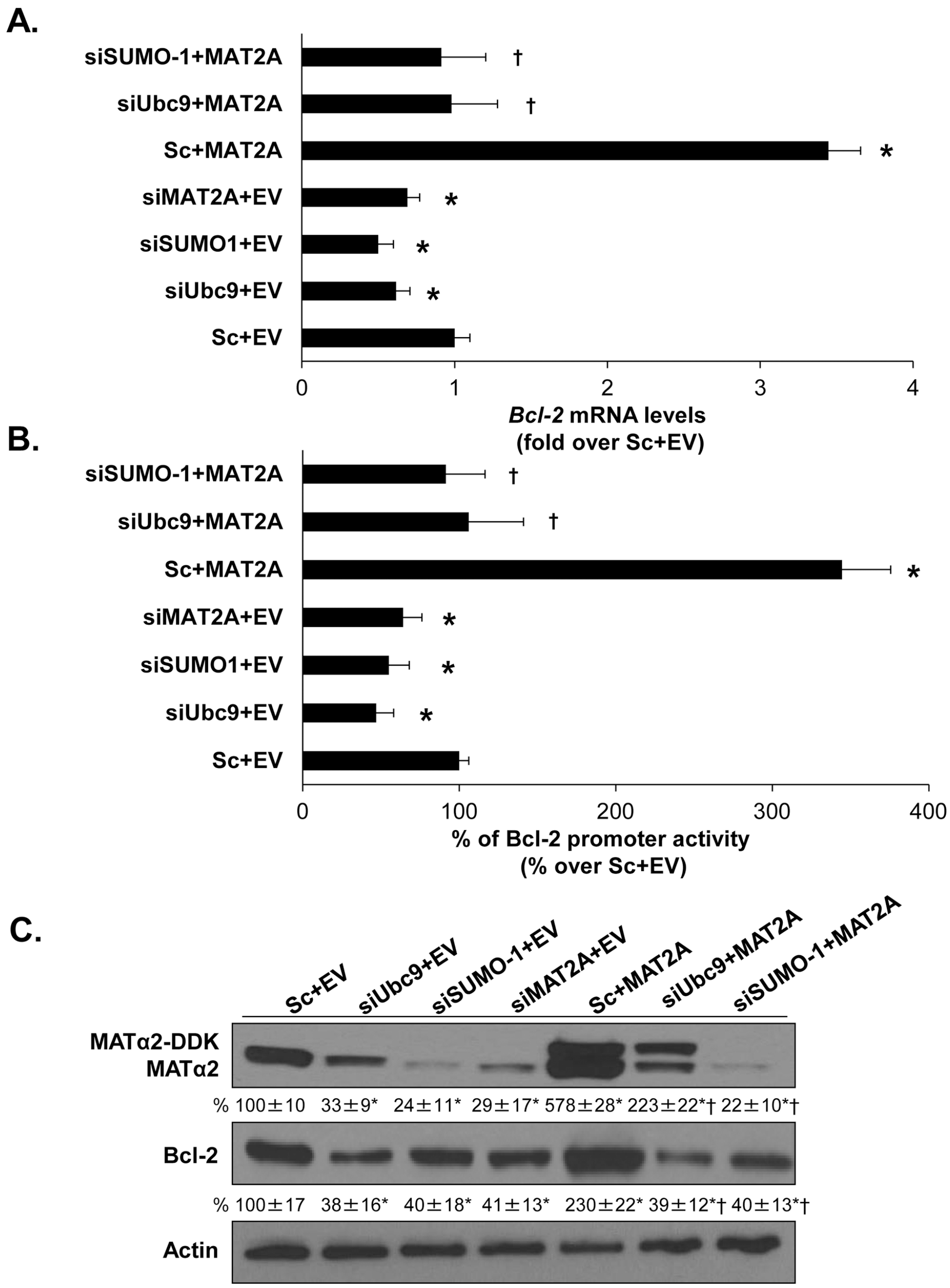

Supplementary Figure S1: MAT2A and sumoylation machinery regulate Bcl-2 expression in RKO cells. A. RKO cells were transfected with siMAT2A, siUbc9 and siSUMO-1 (20 nM) or scrambled siRNA (Sc) for 48 hours and/or MAT2A overexpression vector or empty vector (EV) for 24 hours. The mRNA levels of Bcl-2 were compared to Sc+EV using real-time PCR. Results represent mean ± SEM from 3 experiments in duplicates, *$p < 0.04$ vs. Sc+EV control; †$p < 0.04$ vs. Sc+MAT2A overexpression vector. **B.** Following the same treatments as in "A", RKO cells were transfected with the human *Bcl-2* promoter or pBS-basic as described in Methods. The luciferase activity driven by *Bcl-2* promoter was normalized to that of pBS-Basic and expressed as % over Sc+EV. Results represent mean ± SEM from 4 experiments in duplicate, *$p < 0.04$ vs. Sc+EV control; †$p < 0.03$ vs. Sc+MAT2A overexpression vector. **C.** Cells were treated as in 'A' and total cellular protein was subjected to Western blotting with antibodies against MATα2 or Bcl-2. Densitometric changes were normalized to actin. Representative images and densitometric analysis (% mean ± SEM of Sc+EV, indicated below the blots) from 3 experiments in RKO cells are shown. *$p < 0.03$ vs. Sc+EV, †$p < 0.001$ vs. Sc+MAT2A overexpression vector.

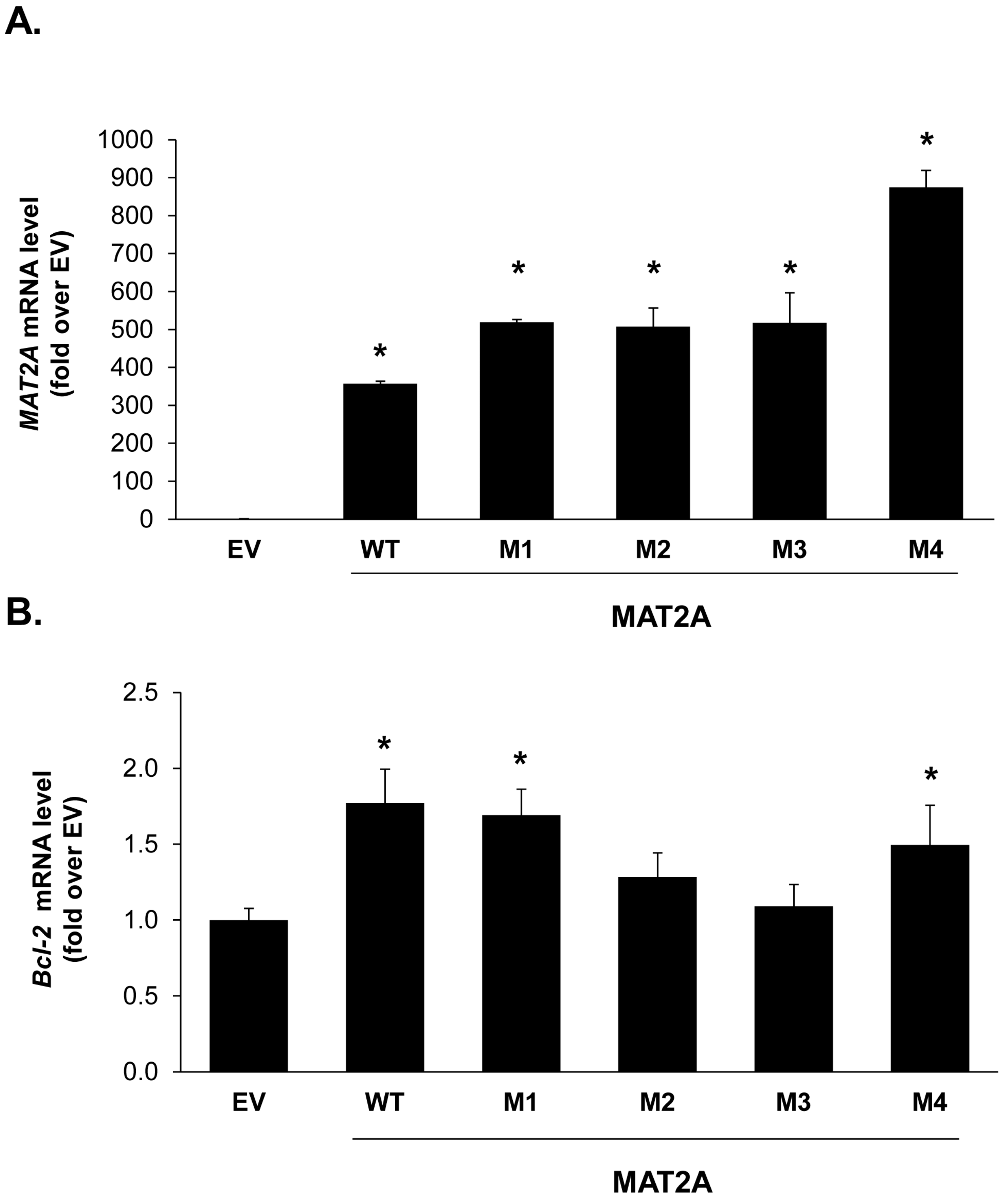

Supplementary Figure S2: Effects of wild-type or mutant MAT2A overexpression on MAT2A and Bcl-2 mRNA levels in RKO cells. RKO cells were transfected with empty vector (EV), hMAT2A-WT, hMAT2A-M1, and hMAT2A-M2, hMAT2A-M3 and hMAT2A-M4 for 24 hours as described in Methods. **A–B.** MAT2A and Bcl-2 expression was determined by real-time PCR. mRNA results are expressed as mean % of EV ± SEM from 3 independent experiments. *$p < 0.01$ vs. EV control for (A), *$p < 0.03$ for (B).

Supplementary Table S1: EMSA primers of Bcl-2 P2 promoter

Probe 1 Fw	TGCGGATTGACATTTCTGTGAA
Probe 1 Rev	TTCACAGAAATGTCAATCCGCA
Probe 2 Fw	GGTTTAATGTAACTTTCAATGG
Probe 2 Rev	CCATTGAAAGTTACATTAAACC
Probe 3 Fw	GGCAGCTTAATACATTCTTTTTAGCCG
Probe 3 Rev	CGGCTAAAAAGAATGTATTAAGCTGCC

BCL-2 P2 PROMOTER SEQUENCE:

GGTTGGGATTCC**TGCGGATTGACA**
TTTCTGTGAAGCAGAAGTCTGGGAATCGATCTGGAAATCCTCCTAATTTT
TACTCCCTCTCCCCGCGACTCCTGATTCATTGGGAAGTTTCAAATCAGCT
ATAACTGGAGAGTGCTGAAGATTGATGGGATCGTTGCCTTATGCATTTGT
TTTGGTTTTACAAAAAGGAAACTTGACAGAGGATCATGCTGTACTTAAAA
AATACAAGTAAGTTCTCTGCACAGGAAATT**GGTTTAATGTAACTTTCAAT**
GGAAACCTTTGAGATTTTTTACTTAAAGTGCATTCGAGTAAATTTAATTT
CCA**GGCAGCTTAATACATTCTTTTTAGCCG**TGTTACTTGTAGTGTGTATG
CCCTGCTTTCACTCAGTGTGTACAGGGAAACGCACCTGATTTTTTACTTA
TTAGTTTGTTTTTTCTTTAACCTTTCAGCATCACAGAGGAAGTAGACTGA
TATTAACAATACTTACTAATAATAACGTGCCTCATGAAATAAAGATCCGA
AAGGAATTGGAATAAAAATTTCCTGCATCTCATGCCAAGGGGGAAACACC
AGAATCAAGTGTTCCGCGTGATTGAAGACACCCCCTCGTCCAAGAATGCA
AAGCACATCCAATAAAATAGCTGGATTATAACTCCTCTTCTTTCTCTGGG
GGCCGTGGGGTGGGAGCTGGGGCGAGAGGTGCCGTTGGCCCCCGTTGCTT TTCCTCTGGGAAGG**ATG**

Supplementary Table S2: Site-directed mutagenesis primers of MATα2 sumoylation sites

MAT2A mut 1 Fw	TTGGGTGGCAAAATCCCTTGTTA**G**AGGAGGTCTGTG
MAT2A mut 1 Rev	CACAGACCTCCT**C**TAACAAGGGATTTTGCCACCCAA
MAT2A mut 2 Fw	CATTATGGTACCTCTCAGA**G**GAGTGAGAGAGAGCTATTA
MAT2A mut 2 Rev	TAATAGCTCTCTCTCACTC**C**TCTGAGAGGTACCATAATG
MAT2A mut 3 Fw	TCAGGGATCTGGATCTGA**G**GAAGCCAATTTATCAGAG
MAT2A mut 3 Rev	CTCTGATAAATTGGCTTC**C**TCAGATCCAGATCCCTGA
MAT2A mut 4 Fw	AGTGCCCAAAAAGCTTA**G**ATATACGCGTACGCGGC
MAT2A mut 4 Rev	GCCGCGTACGCGTATAT**C**TAAGCTTTTTGGGCACT
